# Cardiovascular Reasons for Access to a Tertiary Oncological Emergency Service: The CARILLON Study

**DOI:** 10.3390/jcm12030962

**Published:** 2023-01-26

**Authors:** Jacopo F. Imberti, Anna Maisano, Francesca Rampini, Melania Minnocci, Filippo Bertuglia, Marta Mantovani, Benedetta Cherubini, Davide A. Mei, Leonardo Ferrara, Niccolò Bonini, Anna Chiara Valenti, Marco Vitolo, Giuseppe Longo, Giuseppe Boriani

**Affiliations:** 1 Cardiology Division, Department of Biomedical, Metabolic and Neural Sciences, University of Modena and Reggio Emilia, Policlinico di Modena, Via del Pozzo 71, 41125 Modena, Italy; 2 Clinical and Experimental Medicine PhD Program, University of Modena and Reggio Emilia, 41125 Modena, Italy; 3 Medical Oncology, Azienda Ospedaliera Universitaria di Modena, 41125 Modena, Italy

**Keywords:** acute cardiovascular care, atrial fibrillation, cancer, cardio-oncology, emergency department

## Abstract

Background: The prevalence of acute cardiovascular diseases (CVDs) in cancer patients is steadily increasing and represents a significant reason for admission to the emergency department (ED). Methods: We conducted a prospective observational study, enrolling consecutive patients with cancer presenting to a tertiary oncological ED and consequently admitted to the oncology ward. Two groups of patients were identified based on main symptoms that lead to ED presentation: symptoms potentially related to CVD vs. symptoms potentially not related to CVD. The aims of the study were to describe the prevalence of symptoms potentially related to CVD in this specific setting and to evaluate the prevalence of definite CV diagnoses at discharge. Secondary endpoints were new intercurrent in-hospital CV events occurrence, length of stay in the oncology ward, and mid-term mortality for all-cause. Results: A total of 469 patients (51.8% female, median age 68.0 [59.1–76.3]) were enrolled. One hundred and eighty-six out of 469 (39.7%) presented to the ED with symptoms potentially related to CVD. Baseline characteristics were substantially similar between the two study groups. A discharge diagnosis of CVD was confirmed in 24/186 (12.9%) patients presenting with symptoms potentially related to CVD and in no patients presenting without symptoms potentially related to CVD (*p* < 0.01). During a median follow-up of 3.4 (1.2–6.5) months, 204 (43.5%) patients died (incidence rate of 10.1 per 100 person/months). No differences were found between study groups in terms of all-cause mortality (hazard ratio [HR]: 0.85, 95% confidence interval [CI] 0.64–1.12), new in-hospital CV events (HR: 1.03, 95% CI 0.77–1.37), and length of stay (*p* = 0.57). Conclusions: In a contemporary cohort of cancer patients presenting to a tertiary oncological ED and admitted to an oncology ward, symptoms potentially related to CVD were present in around 40% of patients, but only a minority were actually diagnosed with an acute CVD.

## 1. Introduction

Cardiovascular diseases (CVDs) and cancer are among the leading causes of death worldwide [1]. In the last 30 years, cancer-related mortality steadily declined, while the prevalence of CVDs and cardiovascular deaths in cancer survivors raised [2]. Recent data showed that for women who survived 5 years or more after diagnosis of breast cancer, CVDs exceeded cancer as the leading cause of death [3]. Similarly, several studies pointed out that cancer survivors are at higher risk of developing coronary artery disease (CAD), valvular heart disease (VHD), and heart failure (HF) at long-term follow-up [3,4]. Physicians will have to manage an increasing number of cancer patients presenting with chronic and acute CVDs in the near future [5,6,7]. In this context, an integrated and personalized approach to patient care, relying on multidisciplinary teams including cardiologists and oncologists, is gaining actual relevance [8,9,10], even in the peculiar setting of emergency departments (EDs) [11,12]. Few studies tried to characterize cardiovascular presentations to EDs and associated outcomes among cancer patients and evidence are still sparse, despite the importance of CVD in cancer patients [5,13].

The present study aims to describe the prevalence of symptoms potentially related to CVDs, the prevalence CVD confirmed diagnoses, and mid-term outcomes of cancer patients presenting to a tertiary oncological ED. In our institution, an oncological ED has been active since 2001. It is managed by oncologists and admits all patients with a current or past diagnosis of cancer with acute events requiring medical assessment. 

## 2. Materials and Methods

### 2.1. Study Design and Population

The CARILLON study (Cardiovascular risk and events during follow-up in oncological patients) is an ongoing prospective observational study enrolling consecutive patients with cancer at the Modena University Hospital conducted by the Cardiology Division and the Oncology Department of the University of Modena and Reggio Emilia. For the purpose of this analysis, we included oncological patients presenting to the tertiary oncology ED of the Modena University Hospital and admitted to the oncology ward between 1 September 2021 and 30 September 2022. Exclusion criteria were as follows: (i) age < 18 years, (ii) same day ED discharge without admission to the oncology ward, (iii) positive molecular test for COVID-19 (all patients underwent testing in ED according to our institutional protocols and in case of positive test patients were admitted to specialized COVID-19 wards), and (iv) unwillingness to provide informed consent. In our institution, the oncological ED is distinct from the general ED, but they share some similar pathways for patient management and have equal access to hospital facilities and other specialties. All the patients with a history of cancer or active cancer followed by our Oncology Department are aware that they can refer to the oncology ED for any acute illness in order to receive timely multidisciplinary management.

Cancer types at presentation were classified into 12 categories (Supplemental Table S1) and cancer status was defined as active whenever cancer was diagnosed within the previous 6 months, was recurrent, regionally advanced, or metastatic, or when anticancer treatment was administered within 6 months, or in the case of hematological cancer that was not in complete remission [14]. 

**Figure 1:**
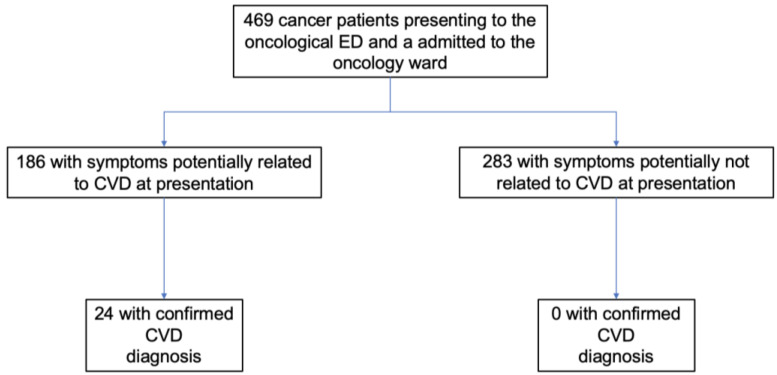
Flow diagram of study design. CVD, cardiovascular disease; ED, emergency department.

Two groups of patients were identified based on the main symptoms/reasons that lead to ED presentation and subsequent hospital admission: symptoms potentially related to CVD vs. symptoms potentially not related to CVD. In the case of multiple symptoms, the presenting symptom was defined as the one that primarily caused the patient to visit the ED (Figure 1).

Symptoms potentially related to CVD included the following: dyspnea, dizziness/syncope, palpitations, fever, chest pain, hypertension/hypotension, and peripheral edema. Potential non-CVD-related symptoms categories were as follows: gastrointestinal (GI), neurological, urinary symptoms, clinical deterioration, pain, fatigue associated with laboratory test alterations, and others (Supplemental Table S2). Thereafter, we identified two groups of patients according to hospital discharge diagnosis (after all diagnostic tests/investigations that were needed according to usual good clinical practice), confirmed diagnosis of CVD (according to current guidelines (Supplemental Table S3)), or diagnosis of other, non-CV pathology at discharge.

### 2.2. Data Collection and Study Outcomes

Data were obtained from electronic and paper medical records and reported on a prespecified, anonymized database. Data collection included: patients’ demographics, CV risk factors, main CV and non-CV comorbidities, cancer type and status, pharmacological treatments at home, symptoms at ED admission, and heart rhythm at the first ECG performed in the ED. Follow-up data included: new intercurrent in-hospital CV events (acute coronary syndromes, brady or tachyarrhythmias, heart failure, hypertension/hypotension, pulmonary embolism, stroke), length of stay in the oncology ward, discharge diagnosis, and all-cause death. The primary objectives of the present observational study were to describe the prevalence of symptoms potentially related to CVD in the specific setting of a tertiary oncological ED and to evaluate the prevalence of definite CV discharge diagnoses in this acute oncological cohort. Only the primary discharge diagnosis was reported for the purpose of the present analysis. As a secondary endpoint, we evaluated new intercurrent in-hospital CV events prevalence, length of stay in the oncology ward, and mid-term mortality for all-cause. 

The present study was approved by the local Institutional Review Boards/Ethics Committee in compliance with national regulations.

### 2.3. Statistical Analysis

Continuous variables were expressed as median with interquartile range (IQR). Categorical variables were expressed as counts and percentages. Between groups, comparisons were made using a chi-square test or Fisher’s exact test (if any expected cell count was less than five) for categorical variables and Mann–Whitney U test for continuous variables. Cumulative survival was evaluated with the use of Kaplan–Meier estimates and results were compared with the log-rank test. Incidence rates were calculated by dividing the number of patients reaching the outcome by the total number of person/months. A Cox-univariate regression analysis was performed to evaluate the association between symptoms potentially related to CVD and study outcomes. Results were expressed as hazard ratio (HR), 95% confidence interval (CI), and *p*-value. A *p*-value < 0.05 was considered statistically significant in all the analyses. Analyses were performed using SPSS^®^ version 26 (IBM Corp., Armonk, NY, USA). 

## 3. Results

### 3.1. Study and Population Characteristics

A total of 469 patients (51.8% female, median age 68.0 (59.1–76.3)) were included in the present analysis. Among cancer types, hematological neoplasms were the most represented (28.3%), followed by GI tract (23.4%), lung (15.9%), and breast (11.5%) cancers (Supplemental Figure S1). One hundred and eighty-six out of 469 (39.7%) patients presented to the ED with symptoms potentially related to CVD (according to evaluations performed in ED). These patients had more often history of atrial fibrillation (AF) as compared to patients presenting to ED without symptoms potentially related to CVDs (16.8% vs. 8.2%, *p* < 0.01). As expected, oral anticoagulants were more often prescribed in the former group (28.7% vs. 15.1%, *p* < 0.01). Otherwise, age, CV risk factors, comorbidities, cardiac implantable electronic devices, and CV medications did not significantly differ between groups. A detailed description of patients’ baseline characteristics is reported in Table 1.

**Figure 2:**
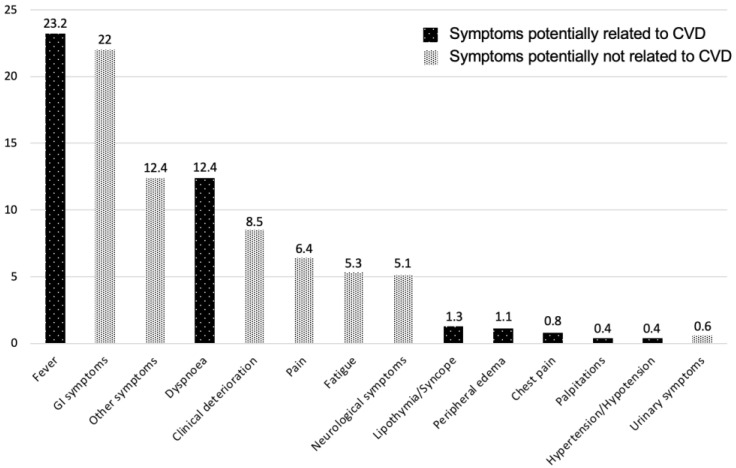
Distribution of presenting symptoms in the emergency department. CVD, cardiovascular disease; GI, gastrointestinal.

The most common presenting symptoms were fever (23.2%), GI symptoms (22.0%), and dyspnea (12.4%) (Figure 2).

### 3.2. Study Outcomes

A discharge diagnosis of CVD was confirmed in 24/186 (12.9%) patients presenting with symptoms potentially related to CVD. On the other hand, no diagnosis of CVD was observed in patients presenting without symptoms potentially related to CVD (*p* < 0.01). Among confirmed CVD diagnoses, de novo HF was the most prevalent (8/24 patients), followed by pulmonary embolism (7/24 patients) and cardiac tamponade (3/24 patients). Other CVD diagnoses are reported in Supplemental Table S4. Presenting symptoms potentially related to CVD had a high sensitivity (100%) and negative predictive value (100%) for subsequent confirmed CVD diagnosis at discharge, but they had modest specificity (63.6%) and positive predictive value (12.9%). Sensitivity, specificity, positive and negative predictive value of potential CVD symptoms are reported in Supplemental Table S5.

**Figure 3:**
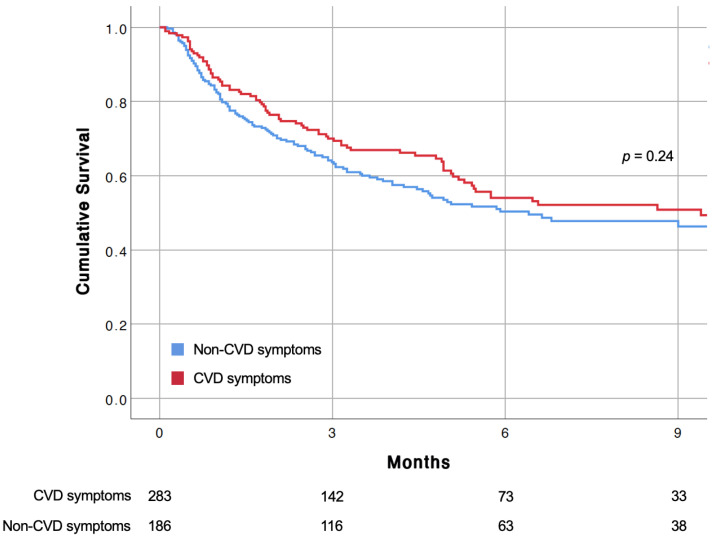
Cumulative incidence of all-cause death according to symptoms at presentation estimated using the Kaplan–Meier method. CVD, cardiovascular disease.

During a median follow-up of 3.4 (IQR 1.2–6.5) months, 204 (43.5%) patients died. The overall incidence rate was 10.1 per 100 person/months (204 first events over 2014.33 person/months). Kaplan–Meier analysis for this outcome showed no significant difference between the two study groups (Figure 3), and this finding was also confirmed by Cox-regression analysis (HR: 0.85, 95% CI 0.64–1.12) (Table 2). New intercurrent in-hospital CV events were detected in 15/186 (8.1%) patients presenting with symptoms potentially related to CVD and 17/283 (6.0%) patients presenting without symptoms potentially related to CVD. Presentation with symptoms potentially related to CVD was not associated with the occurrence of new intercurrent in-hospital CV events (HR: 1.03, 95% CI 0.77–1.37) (Table 2). A detailed description of new intercurrent in-hospital CV events is presented in Supplemental Table S4. No new intercurrent in-hospital CV event led to a primary discharge diagnosis of definite CVD. The median length of stay in the oncology ward was 12 (8–19) days, and it did not differ between study groups (*p* = 0.57).

## 4. Discussion

Our study describes the impact of symptoms potentially related to acute CVDs on ED presentations and clinical outcomes in a contemporary cohort of cancer patients. Our main findings were as follows: (i) almost 40% of patients presenting to the ED who were then admitted to the oncology ward had symptoms potentially related to CVD, but in only a minority of them (13%) was a diagnosis of acute CVD confirmed; (ii) no acute CVD was observed in patients presenting without symptoms potentially related to CVD; (iii) mid-term mortality was substantially high, and did not differ between the two study groups.

Cancer patients are at increased risk of acute CVDs, which require a multidisciplinary management, and a new cancer diagnosis is associated with higher CV death and morbidity [15,16]. Current European Society of Cardiology (ESC) guidelines and consensus documents highlight the importance of a patient-centered approach, including a careful evaluation of cancer-specific treatments and prognosis [5,11,17]. Indeed, the creation of cancer-specific EDs, directly connected either to cancer hospitals or to general hospitals, has been recently advocated. Our study enrolled patients in the unique setting of a tertiary oncological ED linked to an oncology ward, giving a precise real-world picture of admission causes in cancer patients with a specific focus on CVDs. Almost half of our cohort presented to the oncology ED (and were subsequently admitted to the oncology ward) complaining of symptoms potentially related to CVD. Our data highlight that these symptoms are a major cause of presentation to the ED and hospitalization, and specialists should be aware of it. Moreover, a close collaboration between cardiologists and oncologists, even in the early stages of the management of acute cancer patients, is of paramount importance to provide an accurate and timely differential diagnosis. Few studies evaluated the type and prevalence of symptoms in EDs and associated outcomes. A meta-analysis on 18 studies showed that cancer patients experienced a myriad of symptoms, with the most common being pain, respiratory distress, and fever. Over half of ED visits resulted in hospital admission. The authors concluded that inconsistency in cancer symptom reporting was an important gap in knowledge [18]. Our study focused on clinically relevant symptoms that required hospitalization (by physician judgment) and we defined them according to current guidelines whenever possible. Moreover, unlike most studies in literature, we included patients presenting to an oncological ED, which has dedicated personnel and specific expertise in the management of acute cancer patients as compared to general EDs. In our study, symptoms potentially related to CVD showed high sensitivity and negative predictive value for subsequent confirmed CVD diagnosis at discharge, but modest specificity. These findings should be interpreted in light of the specific diagnoses that were recorded at discharge, after a complete clinical workup performed in accordance with good clinical practice. 

Cancer and CVDs interact at multiple levels, sharing risk factors, several common epidemiological and pathophysiological features, and possible detrimental effects of specific treatments [11,19,20,21,22]. It has been hypothesized that cancer survivors have a reduced cardiovascular reserve and higher risk of incident CVDs and CV death as a consequence of multiple sequential or concurrent events, including cancer treatments, CVD risk factors, lifestyle factors, and psychological distress [23]. Chow et al. [24] retrospectively evaluated 1491 patients who had survived 2 years or longer after hematopoietic stem cell transplantation and compared them with frequency-matched people randomly selected from a US database of drivers’ license files. Transplant recipients showed a significantly higher rate of CV mortality (adjusted incidence rate difference, 3.6 per 1000 person/years (95% CI, 1.7–5.5)) and increased cumulative incidence of cardiovascular-related outcomes (e.g., ischemic heart disease, cardiomyopathy or heart failure, stroke, vascular diseases, rhythm disorders, hypertension, renal disease, dyslipidemia, and diabetes) [24]. In our cohort, about 13% of patients were discharged with a confirmed acute CVD and another 6.8% developed new intercurrent in hospital CV-events. Of note, no patient presenting without symptoms potentially related to CVD was discharged with a confirmed acute CVD diagnosis. Thus, it can be speculated that symptoms at presentation may be an important factor when deciding to involve cardiologists in the evaluation of these patients. Moreover, we found that mid-term mortality was substantial (43.5%). Unfortunately, the relatively limited sample size did not allow us to assess the impact of CVDs on patient outcomes. Previous studies reporting on cancer-related ED visits focused on admission rates and cancer types [25,26]. We believe that, in light of the increasing prevalence of acute CVDs in cancer patients, more studies should focus specifically on this subset of patients aiming at better patient characterization [11,27]. It has been demonstrated that an individualized approach to patient care has proven benefits, even in the ED [28], and the better the characterization of patients, the more favorable the outcomes in multiple settings [29,30,31,32]. 

### Study Limitations

This cohort represents a single center experience with a relatively small sample size (which is the result of around 1 year of activity of the oncological ED) which may limit the generalizability of the results. The relatively short follow-up period could have limited the ability to observe significant differences in terms of mortality between the two study groups. However, the high observed mortality rates partially limited the possibility to obtain long-term follow-up. All patients included in the present analysis presented to the oncological ED and were admitted to the oncology ward. Thus, this population may be at higher risk as compared to patients discharged directly from the ED on the same day of the admission. Nevertheless, since symptoms potentially related to CVD are highly clinically relevant, it could be hypothesized that the majority of patients presenting to the ED for potentially CV causes were included. We recognize that the interpretation of symptoms has a certain degree of variability, both from the patient’s and physician’s perspective, but they have been categorized in the most standardized way possible, in accordance with the guidelines. Given the specific setting of this study, it was not possible to gather granular data enabling a deeper characterization of these patients. In particular, it was not possible to collect cancer-specific treatments, which might have impacted on cardiovascular outcomes and novel markers of subclinical cardiac injury [33]. Further studies are needed to better describe the cancer population presenting to EDs, to find potential predictors of CVD, and to identify subpopulations in which ED oncologists and cardiologists can work together. Our study was performed between September 2021 and September 2022 and was therefore conditioned by the fourth and fifth waves of COVID-19 in Italy [34] which, although less severe than the first three waves, deeply affected the clinical landscape and healthcare resources allocation [35,36,37,38,39].

## 5. Conclusions

In a contemporary cohort of cancer patients presenting to a tertiary oncological ED and admitted to an oncology ward, symptoms potentially related to CVD were considerably prevalent, but a minority of patients were diagnosed with an acute CVD.

## Figures and Tables

**Table 1:** Baseline characteristics of study population according to symptoms at presentation.

	Total Cohort (n = 469)	Potential CVD Symptoms (n = 186)	Potential Non-CVD Symptoms (n = 283)	*p*-Value
Female sex, n (%)	243/469 (51.8)	89/186 (47.8)	154/283 (54.4)	0.16
Age (years), median (IQR)	68.0 (59.1–76.3)	69.3 (59.9–77.6)	69.0 (59.4–75.9)	0.49
Caucasian race, n (%)	457/469 (97.4)	181/186 (97.3)	276/283 (97.5)	1.00
BMI (Kg/m^2^), median (IQR)	25.0 (22.0–28.8)	25.1 (22.0–28.9)	25.0 (22.0–28.8)	0.97
BSA (m^2^), median (IQR)	1.8 (1.6–1.9)	1.8 (1.6–2.0)	1.8 (1.7–1.9)	0.26
Hematologic cancer, n (%)	133/469 (28.4)	78/186 (41.9)	55/283 (19.4)	<0.01
Active cancer, n (%)	469/469 (100)	186/186 (100)	283/283 (100)	-
CV risk factors				
Hypertension, n (%)	259/467 (55.5)	107/184 (58.2)	152/283 (53.7)	0.35
DM, n (%)	98/466 (21.0)	42/184 (22.8)	56/282 (19.9)	0.44
Dyslipidemia, n (%)	133/466 (28.5)	60/184 (32.6)	73/282 (25.9)	0.17
Smoking				
Never, n (%)	293/443 (66.1)	119/175 (68.0)	174/268 (64.9)	0.75
Former, n (%)	99/443 (22.3)	38/175 (21.7)	61/268 (22.8)	
Active, n (%)	51/443 (11.5)	18/175 (10.3)	33/268 (12.3)	
Comorbidities				
IHD, n (%)	45/464 (9.7)	18/184 (9.8)	27/281 (9.6)	0.95
CABG, n (%)	8/465 (1.7)	4/184 (2.2)	4/281 (1.4)	0.72
PCI, n (%)	32/465 (6.9)	11/184 (6.0)	21/281 (7.5)	0.53
COPD, n (%)	38/457 (8.3)	16/182 (8.8)	22/275 (8.0)	0.76
PAD, n (%)	54/464 (11.6)	21/183 (11.5)	33/281 (11.7)	0.93
AF/AFL, n (%)	54/466 (11.6)	31/184 (16.8)	23/282 (8.2)	<0.01
CIED				
No CIED, n (%)	452/465 (97.2)	178/184 (96.7)	274/281 (97.5)	0.15
PM, n (%)	9/465 (1.9)	6/184 (3.3)	3/281 (1.1)	
ICD, n (%)	2/465 (0.4)	0/184 (0.0)	2/281 (0.7)	
CRTD, n (%)	2/465 (0.4)	0/184 (0.0)	2/281 (0.7)	
LVEF, median (IQR)	58 (55–60)	58 (55–60)	60 (55–61)	0.32
Heart rhythm				
SR, n (%)	387/423 (91.5)	146/170 (85.9)	241/253 (95.3)	<0.01
AF, n (%)	29/423 (6.9)	19/170 (11.2)	10/253 (4.0)	
CIED-induced, n (%)	7/423 (1.6)	5/170 (2.9)	2/253 (0.8)	
Main ECG features				
1st-degree AVB, n (%)	15/425 (3.5)	7/170 (4.1)	8/255 (3.1)	0.60
LBBB, n (%)	6/425 (1.4)	1/170 (0.6)	5/255 (2.0)	0.41
RBBB, n (%)	28/424 (6.6)	7/169 (4.1)	21/255 (8.2)	0.10
CV medications				
Antiplatelets, n (%)	48/453 (10.6)	20/181 (11.0)	28/272 (10.3)	0.80
Anticoagulant, n (%)	93/453 (20.5)	52/181 (28.7)	41/272 (15.1)	<0.01
ACEi/ARB, n (%)	87/453 (19.2)	29/181 (16.0)	58/272 (21.3)	0.16
BB, n (%)	150/453 (33.1)	68/181 (37.6)	82/272 (30.1)	0.10

Legend: ACEi, angiotensin-converting enzyme inhibitors; AF, atrial fibrillation; AFL, atrial flutter; ARB, angiotensin receptor blockers; AVB, atrioventricular block; BB, beta-blockers; BMI, body mass index; BSA, body surface area; CABG, coronary artery bypass graft; CIED, cardiovascular implantable electronic device; COPD, chronic obstructive pulmonary disease; CRTD, cardiac resynchronization therapy defibrillator; CV, cardiovascular; CVD, cardiovascular diseases; DM, diabetes mellitus; ICD, implantable cardioverter defibrillator; IHD, ischemic heart disease; IQR, Interquartile range; LBBB, left bundle branch block; LVEF, left ventricular ejection fraction; PAD, peripheral artery disease; PCI, percutaneous coronary intervention; PM, pacemaker; RBBB, right bundle branch block; SR, sinus rhythm.

**Table 2:** Effect of symptoms at presentation on outcomes.

	Potential CVD Symptoms		Potential Non-CVD Symptoms		HR (95% CI)	*p*
	n/N (%)	Events/ 100 pts-Months	n/N (%)	Events/ 100 pts-Months		
Mortality	80/186 (43.0)	8.9	124/283 (43.8)	11.2	0.85 (0.64–1.12)	0.24
New in-hospital CV events	15/186 (8.1)	1.7	17/283 (6.0)	1.5	1.03 (0.77–1.37)	0.83
	**Days, Median (IQR)**		**Days, Median (IQR)**			
Length of stay	12 (8–18)		12 (7–20)		-	0.57

Legend: CI, confidence interval; CV, cardiovascular; CVD, cardiovascular disease; HR, hazard ratio; IQR, interquartile range.

## Data Availability

The data presented in this study are available on reasonable request from the corresponding author.

## Supplementary Materials

The following supporting information can be downloaded at: https://www.mdpi.com/article/10.3390/jcm12030962/s1, Figure S1: Overview of cancer types; Table S1: Categories of cancer types; Table S2: Definitions of symptoms; Table S3: Definition of cardiac conditions underpinning a diagnosis of cardiovascular disease; Table S4: Type of confirmed cardiovascular disease at discharge and new in-hospital cardiovascular events; Table S5: Sensitivity, specificity, positive predictive value and negative predictive value of symptoms potentially related to cardiovascular disease for subsequent confirmed diagnosis of cardiovascular disease at discharge [40,41,42,43,44,45,46,47,48,49,50,51,52].
