# Supplementary material for: Cardiovascular Reasons for Access to a Tertiary Oncological Emergency Service: The CARILLON Study
Source: J Clin Med. 2023 Jan 26;12(3):962. doi: 10.3390/jcm12030962 (PMC9917995; doi:10.3390/jcm12030962)
Supplement: Supplementary file 1 [file jcm-12-00962-s001.zip › jcm-2101905-supplementary.pdf]

Supplemental Table S1. Categories of cancer types(1).

Cancer type	Definition
Hematological	Leukemia, lymphoma, multiple myeloma, and other less frequent hematologic malignancies
Gastrointestinal tract	Esophagus, stomach, small and large intestine, liver, pancreas, gallbladder, and biliary tract
Lung	Lung cancer
Breast	Both male and female breast cancer
Urinary	Kidney and bladder cancers
Gynecological	Uterus and ovary cancers
Other	Neuroendocrine cancers, mesothelial cancers, cancers of unknown origin.
Head and neck	Pharynx, tonsil, tongue, mouth, larynx, and thyroid
Skin	Melanoma, basal cell carcinoma, and squamous cell carcinoma
Prostate	Prostate cancer
Neurological	All types of glial neoplasm

Supplemental Table S2. Definitions of symptoms.

**Symptom**	**Definition**
Dyspnea	Subjective awareness of difficulty in breathing
Dizziness and syncope	Syncope is defined as TLOC due to cerebral hypoperfusion, characterized by a rapid onset, short duration, and spontaneous complete recovery(2)
Palpitation	Self-reported abnormally rapid or irregular beating of the heart
Fever	Body temperature >38°C(3)
Chest pain	Any type of patient referred chest pain(4)
Hypertension	Office systolic BP values ≥140 mmHg and/or diastolic BP values ≥90 mmHg(5)
Hypotension	BP values <90/60 mmHg, or need of reducing antihypertensive treatment
Peripheral edema	Peripheral swelling/ edema
GI symptoms	Abdominal pain, nausea and vomiting, constipation and diarrhea, dysphagia, jaundice and GI bleedings
Neurological symptoms	Arms' weakness, walking abnormalities and focal neurological deficits
Urinary symptoms	Hematuria, strangury, and urinary retention
Clinical deterioration	In the absence of a definitive standardization of the definition of deterioration in oncologic patients(6), we considered clinical deterioration as symptoms worsening or a reduction in the global perception of the quality of life(7)
Pain	All types of pain (mostly bone and muscle pain) with the exception of chest pain (classified as *per se*) and abdominal pain (which is included in GI symptoms)
Fatigue associated with laboratory tests alteration	Fatigue and anemia, thrombocytopenia, electrolyte disorders, renal and liver dysfunction

BP, blood pressure; GI, gastrointestinal; TLOC, transient loss of consciousness.

Supplemental Table S3. Definition of cardiac conditions underpinning a diagnosis of cardiovascular disease.

Diagnosis	Definition
Atrial fibrillation	A supraventricular tachyarrhythmia with uncoordinated atrial electrical activation and consequently ineffective atrial contraction(8)
Heart failure	A clinical syndrome consisting of cardinal signs and symptoms (breathlessness, ankle swelling, pulmonary crackles, etc.)(9)
Cardiac tamponade	Compression of the heart due to the pericardial accumulation of fluid, pus, blood, clots, or gas as a result of inflammation, trauma, heart rupture or aortic dissection(10)
Pulmonary embolism	A positive CT showing occlusion in any of the pulmonary arteries(11)
Pericarditis	Inflammatory pericardial syndrome diagnosed with at least 2 of the 4 following criteria: (I) pericarditic chest pain; (II) pericardial rubs; (III) new widespread ST-elevation or PR depression on ECG; (IV) pericardial effusion(10)
Reflex syncope	Vasovagal syncope; situational syncope, carotid sinus syndrome; and other non-classical forms(2)
Acute Myocardial infarction	As defined by ESC guidelines for the management of ACS in patients presenting with or without persistent ST-segment elevation(12, 13)

ACS, acute coronary syndrome; CT, computed tomography; ECG, electrocardiogram.

Supplemental Table S4. Type of confirmed cardiovascular disease at discharge and new in-hospital cardiovascular events.

	**Symptoms potentially related to CVD (n=186)**	**Symptoms potentially not related to CVD (n=283)**
Confirmed diagnosis of CVD, n (%)	AMI, 1/186 (0.5)	-
	Cardiac tamponade, 3/186 (1.6)	
	De novo AF, 2/186 (1.1)	
	HF, 8/186 (4.3)	
	Pericarditis, 1/186 (0.5)	
	PE, 7/186 (3.8)	
	Reflex syncope, 2/186 (1.1)	
In-hospital CV complications, n (%)	AMI, 0/186 (0)	AMI, 1/283 (0.3)
	Angina, 0/186 (0)	Angina, 1/283 (0.3)
	Atrial tachyarrhythmias (AF, AFL, focal atrial tachycardia), 9/186 (4.8)	Atrial tachyarrhythmias (AF, AFL, focal atrial tachycardia), 3/283 (1.1)
	HF, 2/186 (0.4)	HF, 1/283 (0.3)
	Hypertension/Hypotension, 2/186 (0.4)	Hypertension/Hypotension, 4/283 (1.4)
	PE, 1/186 (0.5)	PE, 7/283 (2.5)
	Stroke, 1/186 (0.5)	Stroke, 0/283 (0)

AF, atrial fibrillation; AFL, atrial flutter; AMI, acute myocardial infarction; CV, cardiovascular; CVD, cardiovascular diseases; GI, gastrointestinal; HF, heart failure; PE, pulmonary embolism.

Supplemental Table S5. Sensitivity, specificity, positive predictive value and negative predictive value of symptoms potentially related to cardiovascular disease for subsequent confirmed diagnosis of cardiovascular disease at discharge.

	Sensitivity (%)	Specificity (%)	PPV (%)	NPV (%)
CVD symptoms	100	63.6	12.9	100
Dyspnea	66.6	90.6	27.6	98.1
Syncope	8.3	99.1	33. 3	95.2
Palpitations	8.3	100	100	95.3
Fever	8.3	75.9	1.8	93.9
Chest pain	8.3	99.5	50.0	95.3
Hypo/hypertension	0.0	99.5	0.0	94.9
Peripheral edema	0.0	98.9	0.0	94.8

CVD, cardiovascular disease; NPV, negative predictive value; PPV, positive predictive value.

FIGURES

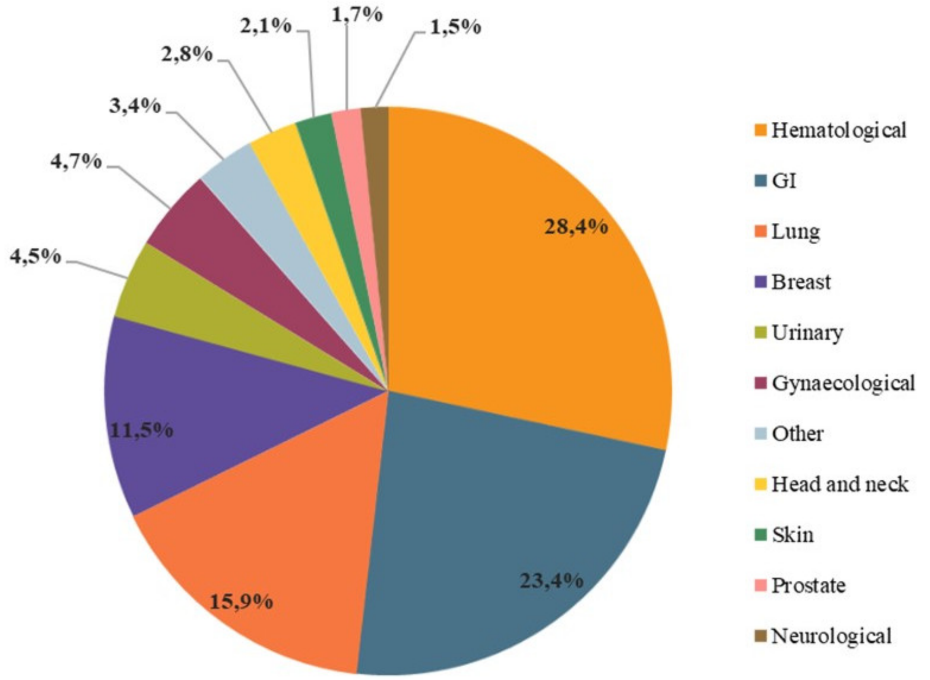

GI, gastrointestinal.

Supplemental Figure S1. Cancer type distribution in the overall cohort.

References

1. Dyba T, Randi G, Bray F, Martos C, Giusti F, Nicholson N, et al. The European cancer burden in 2020: Incidence and mortality estimates for 40 countries and 25 major cancers. Eur J Cancer. 2021;157:308-47.
2. Brignole M, Moya A, de Lange FJ, Deharo JC, Elliott PM, Fanciulli A, et al. 2018 ESC Guidelines for the diagnosis and management of syncope. Eur Heart J. 2018;39(21):1883-948.
3. O'Grady NP, Barie PS, Bartlett JG, Bleck T, Carroll K, Kalil AC, et al. Guidelines for evaluation of new fever in critically ill adult patients: 2008 update from the American College of Critical Care Medicine and the Infectious Diseases Society of America. Crit Care Med. 2008;36(4):1330-49.

4. Erhardt L, Herlitz J, Bossaert L, Halinen M, Keltai M, Koster R, et al. Task force on the management of chest pain. Eur Heart J. 2002;23(15):1153-76.
5. Williams B, Mancia G, Spiering W, Agabiti Rosei E, Azizi M, Burnier M, et al. 2018 ESC/ESH Guidelines for the management of arterial hypertension. Eur Heart J. 2018;39(33):3021-104.
6. Charton E, Cuer B, Cottone F, Efficace F, Touraine C, Hamidou Z, et al. Time to deterioration in cancer randomized clinical trials for patient-reported outcomes data: a systematic review. Qual Life Res. 2020;29(4):867-78.
7. Aaronson NK, Ahmedzai S, Bergman B, Bullinger M, Cull A, Duez NJ, et al. The European Organization for Research and Treatment of Cancer QLQ-C30: a quality-of-life instrument for use in international clinical trials in oncology. J Natl Cancer Inst. 1993;85(5):365-76.
8. Hindricks G, Potpara T, Dagres N, Arbelo E, Bax JJ, Blomström-Lundqvist C, et al. 2020 ESC Guidelines for the diagnosis and management of atrial fibrillation developed in collaboration with the European Association for Cardio-Thoracic Surgery (EACTS): The Task Force for the diagnosis and management of atrial fibrillation of the European Society of Cardiology (ESC) Developed with the special contribution of the European Heart Rhythm Association (EHRA) of the ESC. Eur Heart J. 2021;42(5):373-498.
9. McDonagh TA, Metra M, Adamo M, Gardner RS, Baumbach A, Böhm M, et al. 2021 ESC Guidelines for the diagnosis and treatment of acute and chronic heart failure. Eur Heart J. 2021;42(36):3599-726.
10. Adler Y, Charron P, Imazio M, Badano L, Barón-Esquivias G, Bogaert J, et al. 2015 ESC Guidelines for the diagnosis and management of pericardial diseases: The Task Force for the Diagnosis and Management of Pericardial Diseases of the European Society of Cardiology (ESC)Endorsed by: The European Association for Cardio-Thoracic Surgery (EACTS). Eur Heart J. 2015;36(42):2921-64.
11. Konstantinides SV, Meyer G, Becattini C, Bueno H, Geersing GJ, Harjola VP, et al. 2019 ESC Guidelines for the diagnosis and management of acute pulmonary embolism developed in collaboration with the European Respiratory Society (ERS). Eur Heart J. 2020;41(4):543-603.
12. Collet JP, Thiele H, Barbato E, Barthélémy O, Bauersachs J, Bhatt DL, et al. 2020 ESC Guidelines for the management of acute coronary syndromes in patients presenting without persistent ST-segment elevation. Eur Heart J. 2021;42(14):1289-367.
13. Ibanez B, James S, Agewall S, Antunes MJ, Bucciarelli-Ducci C, Bueno H, et al. 2017 ESC Guidelines for the management of acute myocardial infarction in patients presenting with ST-segment elevation: The Task Force for the management of acute myocardial infarction in patients presenting with ST-segment elevation of the European Society of Cardiology (ESC). Eur Heart J. 2018;39(2):119-77.
